# Early steroid pulse therapy for children with suspected acute encephalopathy: An observational study

**DOI:** 10.1097/MD.0000000000026660

**Published:** 2021-07-30

**Authors:** Yusuke Ishida, Masahiro Nishiyama, Hiroshi Yamaguchi, Kazumi Tomioka, Hiroki Takeda, Shoichi Tokumoto, Daisaku Toyoshima, Azusa Maruyama, Yusuke Seino, Kazunori Aoki, Kandai Nozu, Hiroshi Kurosawa, Ryojiro Tanaka, Kazumoto Iijima, Hiroaki Nagase

**Affiliations:** a Department of Pediatrics, Kobe University Graduate School of Medicine, Kobe, Japan; b Department of Neurology, Hyogo Prefectural Kobe Children's Hospital, Kobe, Japan; c Department of Pediatric Critical Care Medicine, Hyogo Prefectural Kobe Children's Hospital, Kobe, Japan; d Department of Emergency and General Pediatrics, Hyogo Prefectural Kobe Children's Hospital, Kobe, Japan.

**Keywords:** acute encephalopathy, aspartate aminotransferase, children, steroid pulse

## Abstract

Steroid pulse therapy is widely used to treat virus-associated acute encephalopathy, especially the cytokine storm type; however, its effectiveness remains unknown. We sought to investigate the effectiveness of early steroid pulse therapy for suspected acute encephalopathy in the presence of elevated aspartate aminotransferase (AST) levels.

We enrolled children admitted to Hyogo Children's Hospital between 2003 and 2017 with convulsions or impaired consciousness accompanied by fever (temperature >38°C). The inclusion criteria were: refractory status epilepticus or prolonged neurological abnormality or hemiplegia at 6 hours from onset, and AST elevation >90 IU/L within 6 hours of onset. We excluded patients with a neurological history. We compared the prognosis between the groups with or without steroid pulse therapy within 24 hours. A good prognosis was defined as a Pediatric Cerebral Performance Category Scale (PCPC) score of 1-2 at the last evaluation, within 30 months of onset. Moreover, we analyzed the relationship between prognosis and time from onset to steroid pulse therapy.

Fifteen patients with acute encephalopathy and 5 patients with febrile seizures were included in this study. Thirteen patients received steroid pulse therapy within 24 hours. There was no between-group difference in the proportion with a good prognosis. There was no significant correlation between PCPC and timing of steroid pulse therapy (rs = 0.253, *P* = .405). Even after excluding 2 patients with brainstem lesions, no significant correlation between PCPC and steroid pulse therapy timing (rs = 0.583, *P* = .060) was noted. However, the prognosis tended to be better in patients who received steroid pulse therapy earlier.

Steroid pulse therapy within 24 hours did not improve the prognosis in children with suspected acute encephalopathy associated with elevated AST. Still, even earlier administration of treatment could prevent the possible neurological sequelae of this condition.

## 1 Introduction

Acute encephalopathy is a serious complication of pediatric viral infections, including influenza. More than 300 patients are diagnosed with virus-associated acute encephalopathy every year in Japan, and the risk of mortality and sequelae is high.^[1]^ There are 3 main categories of acute encephalopathy. The first category is caused by metabolic derangement, which consists of various inherited metabolic disorders and the classical Reye syndrome. The second category is characterized by a systemic cytokine storm and vasogenic brain edema, and includes Reye-like syndrome, hemorrhagic shock and encephalopathy syndrome (HSES), and acute necrotizing encephalopathy (ANE).^[2,3]^ The third category is caused by excitotoxicity and includes acute encephalopathy with biphasic seizures and late reduced diffusion (AESD).^[1,2]^ Sequelae of acute encephalopathy are observed in 44% of cases; specifically, sequelae or death was observed in 87% and 90% of ANE and HSES cases, respectively.^[1]^ Several specific treatments for virus-associated acute encephalopathy have been proposed for improving prognosis.^[4]^ Particularly, steroid pulse therapy is widely used for its anti-inflammatory action; however, it has little supporting evidence.^[4,5]^ Okumura et al reported that steroid therapy within 24 hours of onset improved prognosis of ANE without brainstem lesions.^[6]^ In contrast, Hayashi et al reported no correlation between steroid therapy within 48 hours of onset and good outcome in acute encephalopathy with reduced subcortical diffusion.^[7]^

Previously, we reported 3 risk factors for poor outcome as follows:

(1) refractory status epilepticus (RSE);
(2) prolonged neurological abnormality at 6 hours from onset; and
(3) aspartate aminotransferase (AST) > 90 IU/L within 6 hours of onset.^[8,9]^

Moreover, we have previously assessed early specific treatment according to the aforementioned criteria and found that targeted temperature management (TTM) might be effective in children with suspected acute encephalopathy without AST elevation (>90 IU/L within 6 hours of onset).^[10]^ However, its therapeutic effects in patients with elevated AST remain unknown.

We sought to assess the effectiveness of early steroid pulse therapy. We retrospectively compared patients with suspected acute encephalopathy with AST elevation who had received steroid pulse therapy within 24 hours of onset and those who had not.

## 2 Materials and methods

### 2.1 Subjects

We enrolled pediatric patients admitted to Hyogo Children's Hospital from January 2003 to December 2017 with convulsions or disturbances of consciousness accompanied by fever (temperature > 38°C). The inclusion criteria were as follows:

(1) RSE, which was defined as convulsions longer than 60 minutes refractory to 1 or more anticonvulsants, or
(2) prolonged neurological abnormality defined as a Glasgow Coma Scale score of <15 or hemiplegia at 6 hours from onset, and
(3) AST > 90 IU/L within 6 hours of onset. We excluded patients with a past neurological history (epilepsy, chromosomal abnormality, brain hemorrhage, hydrocephalus, history of intracranial surgery, or intellectual disability), cerebrospinal fluid cell counts > 8/μL, trauma, and asphyxia.

### 2.2 Methods

We conducted a historical cohort study using a database. We collected information on patient background, treatment, prognosis, and final diagnosis. We divided the patients into 2 groups based on whether they had received steroid pulse therapy within 24 hours of onset. Next, we conducted between-group comparisons of the clinical characteristics and prognosis. Moreover, we examined the relationship between prognosis and time from onset to starting steroid pulse therapy. The prognosis was determined using the Pediatric Cerebral Performance Category Scale (PCPC)^[11]^ at the last evaluation, within 30 months of onset, with a PCPC score of 1 to 2 and 3 to 6 being defined as good and poor, respectively. Onset was defined as the time of initial recognition of neurological symptoms, including convulsions or impaired consciousness, based on previously determined criteria.^[8,10,12,13]^

Moreover, we collected information on sex, age at onset, convulsion duration, brainstem imaging abnormalities, blood test values within 6 hours of onset, presence or absence of TTM and a mitochondrial drug cocktail,^[14]^ and the number of used anticonvulsants. In case there were no data within 6 hours of onset, the blood test values were considered missing values. We used the initial result in cases where multiple blood tests were performed.

### 2.3 Treatment protocol

We admitted patients to the pediatric intensive care unit and carefully monitored them. Upon obtaining consent from the parents, the patients underwent treatment for acute encephalopathy. In our institute, patients with AST > 90 IU/L have been receiving steroid pulse therapy since November 2009 when we got a preliminary data suggesting AST > 90 IU/L was associated with cytokine storm type acute encephalopathy.^[8]^ Sex, age, convulsion duration, brain stem lesions, laboratory data except AST, and cytokine levels did not influence the decision to initiate steroid pulse therapy. We administered methylprednisolone 30 mg/kg (maximum 1000 mg) as steroid pulse therapy for 3 days; subsequently, we administered oral prednisolone for 4 days. Patients with stable cardiovascular dynamics were intubated and underwent TTM under general anesthesia as previously reported.^[15]^ The targeted temperature of the regimen was 34.5 ± 0.5°C before December 2005 and 36.0 ± 0.5°C after January 2006. Moreover, patients with AST > 90 IU/L underwent a mitochondrial drug cocktail after March 2016.

### 2.4 Statistical analysis

We conducted statistical analyses using Fisher's exact test and Mann-Whitney U test in EZR (Saitama Medical Center, Jichi Medical University, Saitama, Japan), which is a graphical user interface for R (version 3.1.2; The R Foundation for Statistical Computing, Vienna, Austria).^[16]^ The correlations were examined using Spearman's rank correlation coefficient. The statistical significance level was set at *P* < .05. The results were also presented as odds ratios with 95% confidence intervals in the comparison of background characteristics.

### 2.5 Ethics committee

This study was approved by the Ethics Committee of Kobe University Graduate School of Medicine and Kobe Children's Hospital, and was performed in accordance with the Declaration of Helsinki. The need for informed consent was waived due to the design of the observational study.

## 3 Results

### 3.1 Characteristics

A total of 1,077 patients were admitted with convulsions or disturbances of consciousness accompanied by fever. Among them, 42 patients had RSE or prolonged neurological abnormality, as well as AST > 90 IU/L within 6 hours from onset. We excluded 17 patients with a past neurological history, three patients with cerebrospinal fluid cells > 8/μL, one patient with trauma, and 1 patient with asphyxia. Finally, we included 20 patients; among them, 13 received steroid pulse within 24 hours while 7 did not. Among those who did not receive steroid pulse therapy within 24 hours, none received it after 24 hours from onset. All the patients who did not receive steroid pulse therapy were admitted before March 2009 while those who did were admitted after October 2009. Two patients who did not receive steroid pulse therapy were given dexamethasone within 24 hours of onset (Table 1).

**Table 1:** Demographics, clinical course, treatment, and prognosis of all the patients (n = 20).

No.	Age (mo)	Sex	Convulsion Duration (min)	Number of anticonvulsants	Consciousness disturbance at 6 h from onset	RSE	Brainstem imaging abnormalities	Maximum AST within 6 h	Timing of steroid pulse (h)	Dexamethasone within 24 h	TTM	Mitochondrial drug cocktails	Diagnosis	Prognosis (PCPC)	Timing of prognostic evaluation (mo)
Steroid pulse															
1	98	F	0	1	+	−	+	1760	3	−	+	−	HSES	6	0.1
2	17	F	105	4	+	+	−	163	8	−	+	−	Reye-like syndrome	5	12.8
3	27	F	125	3	+	−	−	142	15	−	+	+	AESD	4	22.6
4	1	F	20	2	+	−	+	103	11	−	−	−	Unclassified AE	4	24.2
5	8	M	72	4	+	−	−	116	22	−	+	−	AESD	3	25.6
6	19	F	78	4	+	+	−	159	8	−	+	+	AESD	3	22.5
7	137	M	210	4	+	+	−	150	5	−	+	−	AESD	3	23.4
8	2	M	0	3	+	−	−	247	9	−	+	+	Unclassified AE	3	24.8
9	62	F	211	4	+	+	−	1798	8	−	+	+	HSES	1	12.7
10	41	F	0	0	+	−	−	110	5	−	−	−	MERS	1	24.4
11	10	F	73	3	+	+	−	100	5	−	+	+	FS	1	19.4
12	18	F	125	3	+	+	−	106	6	−	+	−	FS	1	21.3
13	90	F	90	2	−	+	−	107	7	−	−	−	FS	1	18.8
Non-steroid pulse															
1	13	M	310	2	+	−	+	7230		+	−	−	Reye-like syndrome	6	23.9
2	10	M	555	5	+	+	−	137		−	+	−	Unclassified AE	4	23.9
3	19	M	1	4	+	−	−	99		+	+	−	Unclassified AE	4	2.9
4	10	M	2	4	+	−	−	166		−	−	−	HSES	3	25.9
5	4	M	3	4	+	−	−	143		−	+	−	HSES	2	25.4
6	19	F	220	3	+	+	−	127		−	+	−	FS	1	20
7	144	M	152	2	+	+	−	121		−	+	−	FS	1	4.9

With regard to the background characteristics of the patients, there were significantly more girls in the group receiving steroid pulse than those in the group not receiving steroid pulse. There was no significant between-group difference in the age of onset, seizure duration, and brainstem imaging abnormalities. Regarding the initial blood test values, there was a lower platelet count in the group receiving steroid pulse therapy. There was no significant between-group difference in the levels of white blood cells, blood glucose, sodium, AST, lactate dehydrogenase, creatine kinase, creatinine, C-reactive protein, base excess, pH, lactic acid, and maximum AST within 6 hours. Regarding treatment, there was no significant between-group difference in the presence or absence of TTM, a mitochondrial drug cocktail, and the number of anticonvulsants used (Table 2). Cytokine levels were tested in 2 of the 13 patients receiving steroid pulse therapy (No. 1 and 9). Both patients were diagnosed with HSES. The serum levels of interleukin 6, interleukin 10, and interferon gamma were 16601 pg/mL, 2094pg/mL, and 38.8 pg/mL, respectively, in patient No. 1, and 3483 pg/mL, 1004 pg/mL, and 84.4 pg/mL, respectively, in patient No. 9. Cytokine levels were not evaluated for patients who were not undergoing steroid pulse therapy.

**Table 2:** Patients’ background characteristics.

	Steroid pulsen = 13	Non-steroid pulsen = 7	*P*-value	Odds ratio (95% CI)
Sex, male	3 (23%)	6 (86%)	.017	0.05 (0.00–0.44)
Age (months)	19 (10–62)	13 (10–19)	.633	1.01 (0.98–1.03)
Convulsion duration (minutes)	78 (20–125)	152 (2.5–265)	.302	0.99 (0.98–1.00)
Brainstem imaging abnormalities	2 (15%)	1 (14%)	1.000	1.09 (0.09–26.4)
WBC (/μL)	11200 (8100–18300)	18820 (15650–35300)	.097	1.00 (1.00–1.00)
PLT (× 10^4^/μL)	26.3 (17.6–31.8)	37.8 (31.6–49.9)	.030	0.91 (0.80–0.99)
Glu (mg/dL)	132 (87–214)	89 (26–165)	.183	1.01 (0.99–1.02)
Na (mEq/L)	134 (133–138)	137 (135–143)	.141	0.88 (0.70–1.02)
AST (U/L)	110 (100–163)	127 (64–140)	.937	1.00 (0.99–1.00)
LDH (U/L)	571 (404–915)	492 (338–652)	.843	1.00 (1.00–1.00)
CK (U/L)	231 (141–290)	424 (235–668)	.234	0.99 (0.99–1.00)
Cre (mg/dL)	0.35 (0.28–0.61)	0.80 (0.56–0.85)	.302	0.50 (0.04–5.29)
CRP (mg/dL)	0.52 (0.14–1.10)	0.20 (0.01–0.65)	.284	2.63 (0.56–25.1)
BE	−9.6 (−11.1 to −6.5)	−13.6 (−14.3 to −9.1)	.142	1.11 (0.93–1.37)
pH	7.16 (7.08–7.27)	7.19 (7.03–7.24)	.899	1.03 (0.00–431.1)
Lac (mmol/L)	3.2 (2.5–5.1)	3.3 (2.2–7.3)	1.000	0.93 (0.72–1.19)
Maximum AST (U/L) within 6 hours	142 (107–163)	137 (124–155)	.877	1.00 (1.00–1.00)
Targeted temperature management	9 (69%)	5 (71%)	1.000	0.90 (0.10–6.57)
Mitochondrial rescue drugs	5 (38%)	0 (0%)	.114	ND
Number of anticonvulsants	3 (2–3)	4 (2.5–4)	.384	0.63 (0.22–1.42)

### 3.2 Between-group comparisons of the prognosis

The prognosis was good in 5 out of 13 patients (38%) and 3 out of 7 patients (43%) in the groups with and without steroid pulse treatment, respectively; there was no significant between-group difference (Table 3). There was no significant between-group difference in the timing of the final prognostic evaluation within 30 months, with death cases excluded (Table 3). In the group with steroid pulse, three patients were diagnosed with cytokine storm type acute encephalopathy, four with excitotoxicity type acute encephalopathy, three with unclassified acute encephalopathy, and 3 with febrile seizures (FS). In the group without steroid pulse, there were 3 cases of the cytokine storm type, one case of excitotoxicity, one case of unclassified, and 2 cases of FS. Table 3 shows the PCPC scores at the last follow-up within 30 months of onset.

**Table 3:** Prognosis and diagnoses.

	Steroid pulsen = 13	Non-steroid pulsen = 7	*P*-value
Prognosis: Good (PCPC 1-2)	5 (38%)	3 (43%)	1.000
Timing of prognostic evaluation (months)	22.6 (19.3–24.3)	23.9 (12.5–24.7)^∗^	.966
Diagnosis	Acute encephalopathy 10 (77%)Cytokine storm type 3 (23%)AESD 4 (31%)Unclassified 3 (23%)Febrile seizure 3 (23%)	Acute encephalopathy 5 (71%)Cytokine storm type 3 (43%)AESD 1 (14%)Unclassified 1 (14%)Febrile seizure 2 (29%)	

### 3.3 Relationship between prognosis and time to steroid pulse therapy initiation

**Figure 1:**
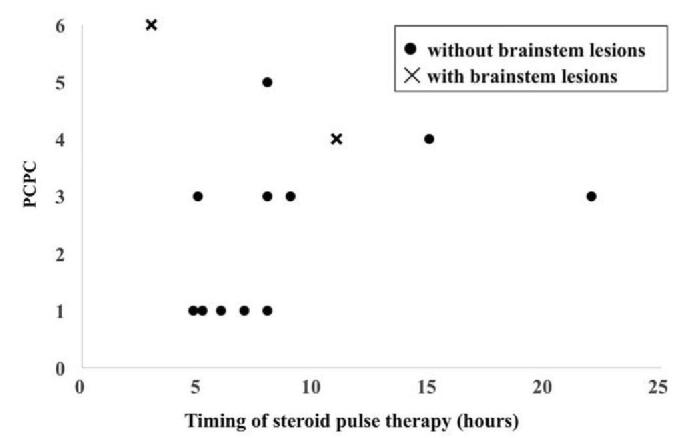
Relationship between the prognosis and timing of steroid pulse therapy in cases of suspected acute encephalopathy. PCPC = pediatric cerebral performance category scale.

Figure 1 represents the relationship between prognosis and the timing of starting steroid pulse therapy. There was no significant correlation between PCPC and timing of steroid pulse therapy (rs = 0.253, *P* = .405). After excluding 2 patients with brainstem lesions, there was no significant correlation between PCPC and timing of steroid pulse therapy (rs = 0.583, *P* = .060). Nevertheless, the trend of the prognosis was better in patients with earlier steroid pulse therapy.

## 4 Discussion

Our findings suggest that steroid pulse therapy is not effective for suspected acute encephalopathy with elevated AST levels in children, despite early treatment induction. Okumura et al reported that 58% of 12 patients with ANE without brainstem lesions who received steroid pulse therapy within 24 hours after onset presented no sequelae or mild sequelae. In contrast, all the patients who did not receive steroid pulse therapy within 24 hours had poor outcome, which indicated the possibility of improving prognosis by early steroid administration.^[6]^ Hayashi et al reported that steroid administration in 73% of children with acute encephalopathy reduced subcortical diffusion (24% received it within 48 hours after onset); however, it was not related to good outcome.^[7]^ Moreover, Takanashi et al reported that early steroid pulse therapy could be effective for encephalopathy secondary to Shiga toxin-producing *Escherichia coli* O111, which involves inflammatory cytokines.^[17]^ These reports indicate that steroid therapy could be effective for cytokine storm type acute encephalopathy.

Previous studies enrolled participants with a confirmed final diagnosis, including ANE or AESD. In contrast, we enrolled participants with suspected acute encephalopathy, which is a combination of symptoms (RSE or persistent disturbance of consciousness with AST elevation) within 6 hours of onset. Therefore, the final diagnosis in our study consisted of not only acute encephalopathy but also FS; furthermore, 6 (30%) patients were diagnosed with cytokine storm type acute encephalopathy. Another unique trait of our study was the early induction of steroid pulse therapy. In this study, steroid pulse therapy was started within 12 hours in the majority of the patients while previous reports started steroid therapy within 24 hours^[6]^ or after several days.^[17]^ Our study included a heterogeneous population in terms of the final diagnosis; however, we included homogenous conditions or symptom combinations at 6 hours from onset. Therefore, it could be more feasible for actual clinical practice given the difficulty in the early differentiation of ANE or AESD from other diseases.

Based on our previous report, we adopted AST > 90 IU/L as the reference value for suspecting cytokine storm type acute encephalopathy.^[8,9]^ Specifically, 23% and 43% of participants in the steroid pulse and no steroid pulse group, respectively, had the cytokine storm type. Compared to our previous study, we employed a much lower positive predictive value of AST > 90 IU/L for the cytokine storm type.^[8]^ We hypothesized that an aggressive intervention, especially steroid pulse administration, could modify the final diagnosis from cytokine storm type to a different clinical picture. Nevertheless, an early AST increase seems to be a predictive factor for cytokine storm type acute encephalopathy since the cytokine type was reported to represent only a total of 7% of virus-associated acute encephalopathy cases in Japan.^[1]^

Other reports on the relationship between acute encephalopathy and AST elevation indicate that AST ≥46 IU/L on admission is an independent prognostic factor (odds ratio 18.5) for poor outcome in acute encephalopathy,^[18]^ while AST elevation on admission in influenza encephalopathy is a predictor of mortality (odds ratio for AST <100: 100–500: >500 = 1: 5.45: 17.38).^[18,19]^ We found that 60% of participants had severe sequelae, which is consistent with previous reports and indicates that AST elevation predicts poor prognosis early in the disease.

Previously, we reported that clinical symptoms were dramatically worsened between a few and 13 hours from onset in a review on fatal acute encephalopathy.^[13]^ Taken together with the above report and previous reports regarding treatment time-windows for various neuro-critical conditions including hypoxia, stroke, status epilepticus, and acute encephalopathy,^[20–25]^ we hypothesize that very early intervention within several hours is needed for neuroprotection in severe acute encephalopathy. Therefore, in this report, we examined the relationship between prognosis and time from onset to starting steroid pulse therapy. Our study did not show effectiveness of steroid pulse therapy within 24 hours, indicating 2 possibilities:

(1) early administration of steroid pulse therapy for neuro-critical conditions (RSE or persistent disturbance of consciousness with AST elevation) is ineffective, or
(2) even earlier intervention is necessary for these neuro-critical conditions.

The poor prognosis of Case 1 in Table 1, who had taken steroid pulse therapy within 3 hours of onset, supports the former possibility. On the other hand, the tendency of a better prognosis with the earlier timing of steroid pulse initiation (Fig. 1) may support the latter. To elucidate on this, it is necessary to accumulate data and investigate the relationship between time from onset to administration and prognosis.

All 3 patients with brainstem lesions had a poor prognosis with or without steroid pulse therapy. This is consistent with a previous report that documented that patients with ANE with brainstem lesions had a poor prognosis regardless of steroid administration.^[6]^

Between-group comparisons of the characteristics indicated that the steroid pulse group had more females, and lower platelet counts. To date, there has been no report on sex-based differences in the diagnosis and prognosis of acute encephalopathy. Regarding the differences in the platelet count, a previous study indicated that levels of <10 × 10^4^/μL were a poor prognostic factor in influenza encephalopathy.^[19]^ However, a previous study reported that the platelet count in acute encephalopathy is higher in the poor prognosis group than in the good prognosis group (median 39.5 × 10^4^/μL vs. 25.1 × 10^4^/μL).^[18]^ In this study, there was only 1 case with a platelet count of <10 × 10^4^/μL in the steroid pulse group, and none in the no steroid pulse group. Therefore, the relationship between a low platelet count and prognosis remains unknown.

Cytokine storm type acute encephalopathy is characterized by systemic organ damage and encephalopathy involving hypercytokinemia, liver damage, and hepatic mitochondrial damage.^[2]^ We tested serum cytokine levels in 2 patients who were both undergoing steroid pulse therapy and diagnosed with HSES. Both patients showed hypercytokinemia and elevated liver enzymes. One patient died, but the other patient had no neurological sequelae. There have been some previous reports of elevated inflammatory cytokines in virus-associated acute encephalopathy.^[26–28]^ On the other hand, HSES cases are diagnosed based on clinical criteria, which do not need proof of hypercytokinemia.^[3,29]^ Another report suggested that cytokine levels in patients with severe acute encephalitis were lower than in patients with febrile seizures.^[30]^ Interestingly, Ito et al reported that some cytokine levels were higher in patients with pneumonia than in those with both pneumonia and encephalopathy in influenza infection.^[31]^ Taken together, cytokines seem to be involved in virus-associated acute encephalopathy; however, a neurological prognosis might be determined by other conditions.

This study has a number of limitations. As previously mentioned, one of the distinctive traits of our study was that our participants initially had homogenous conditions or symptoms but heterogeneous final diagnoses, which should be considered when interpreting our results. Our results do not directly suggest the ineffectiveness of steroid pulse therapy for a single pathology such as cytokine storm type acute encephalopathy. This study had other limitations. First, this was a single-center retrospective study with a small sample size. The small sample size could not justify a stratified analysis or adjusted analysis. Second, the patients who underwent steroid pulse therapy were admitted after October 2009 while those who did not were admitted before March 2009. Therefore, there might be potential between-group differences in the treatments for acute encephalopathy. We basically did not use cytokine levels as a decision-making parameter; however, more severely affected patients with higher cytokine levels might be potentially treated more aggressively, including with steroid pulse therapy. Third, 2 patients received dexamethasone within 24 hours of onset in the no steroid pulse group, which might affect the results. However, given that both patients had a poor prognosis, dexamethasone is unlikely to have affected the conclusions. Fourth, prognosis was evaluated within 6 months in 2 patients in non-steroid pulse group (No. 3 and 7), which limited long-term prognostic evaluation. Finally, our findings cannot be applied to patients without AST elevation (AST ≤ 90 IU/L) within 6 hours of onset.

In conclusion, our findings suggest that steroid pulse therapy within 24 hours of onset does not improve the prognosis in children with suspected acute encephalopathy associated with increased AST. Nevertheless, our findings indicate that initiating treatment even earlier could prevent neurological sequelae. Because there are several important limitations, including a small sample size, further studies are needed in the future to strengthen the evidence presented in this study.

## Acknowledgments

The authors thank all participating physicians and nurses who took care of the patients. They also thank the children and their parents for their kind collaboration. They also thank the Clinical and Translational Research Center of Kobe University for the statistical analysis of the data. They would like to thank Editage (www.editage.com) for English language editing.

## Author contributions

YI and MN designed the project and first drafted the manuscript. AM and HN designed and supervised the project and critically reviewed and revised the manuscript for important intellectual content. HY, KY, and HT revised the manuscript for important intellectual content. ST, DT, YS, and KA collected data and critically revised the article. KN, HK, RT, and KI contributed to data analysis and interpretation, critical revision of the article, and final approval of the version to be published. All authors approved the final manuscript as submitted and agree to be accountable for all aspects of the work.

**Conceptualization:** Yusuke Ishida, Masahiro Nishiyama, Azusa Maruyama, Hiroaki Nagase.

**Data curation:** Yusuke Ishida, Masahiro Nishiyama, Hiroshi Yamaguchi, Kazumi Tomioka, Hiroki Takeda, Shoichi Tokumoto, Daisaku Toyoshima, Yusuke Seino, Kazunori Aoki.

**Formal analysis:** Masahiro Nishiyama, Kandai Nozu, Hiroshi Kurosawa, Ryojiro Tanaka, Kazumoto Iijima.

**Funding acquisition:** Masahiro Nishiyama.

**Investigation:** Masahiro Nishiyama, Hiroshi Yamaguchi, Kazumi Tomioka, Hiroki Takeda, Daisaku Toyoshima, Yusuke Seino, Kazunori Aoki.

**Methodology:** Yusuke Ishida, Masahiro Nishiyama.

**Project administration:** Masahiro Nishiyama, Kazumoto Iijima, Hiroaki Nagase.

**Supervision:** Azusa Maruyama, Hiroaki Nagase.

**Validation:** Hiroshi Yamaguchi, Kazumi Tomioka, Hiroki Takeda, Shoichi Tokumoto, Kandai Nozu, Hiroshi Kurosawa, Ryojiro Tanaka.

**Writing – original draft:** Yusuke Ishida, Masahiro Nishiyama.

**Writing – review & editing:** Hiroshi Yamaguchi, Shoichi Tokumoto, Daisaku Toyoshima, Azusa Maruyama, Yusuke Seino, Kazunori Aoki, Kandai Nozu, Hiroshi Kurosawa, Ryojiro Tanaka, Kazumoto Iijima, Hiroaki Nagase.
